# EVALUATION OF A NURSE PRACTITIONER-LED VIRTUAL BEHAVIORAL MEDICINE PROGRAM: INNOVATIVE NEUROPSYCHIATRIC CARE

**DOI:** 10.1093/geroni/igae098.4377

**Published:** 2024-12-31

**Authors:** Shirin Vellani, Lynn Haslam, Miles Luke, Amaya Singh, Andrea Iaboni

**Affiliations:** Toronto Rehabilitation Institute - University Health Network, Toronto, Ontario, Canada; Toronto Rehabilitation Institute - University Health Network, Toronto, Ontario, Canada; Toronto Rehabilitation Institute - University Health Network, Toronto, Ontario, Canada; Baycrest Health Sciences, Toronto, Ontario, Canada; University of Toronto, Toronto, Ontario, Canada

## Abstract

People living with dementia in long-term care (LTC) who exhibit severe neuropsychiatric symptoms are often at risk of being transferred to the hospital from their familiar environment. The virtual Behavioral Medicine (VBM) model was designed to manage these symptoms with intensive virtual support within LTC to prevent hospitalization. We adapted this model to a nurse practitioner (NP)-led approach and conducted a program evaluation to assess its implementation and preliminary effectiveness during its first year. Between April 2023 to March 2024, 102 patients were referred for management of severe neuropsychiatric symptoms and/or consideration for admission to our psychogeriatric unit. The average wait time from referral to the first consultation was 10 days, with follow-ups occurring every 7.6 days by an NP and every 16.2 days involving a geriatric psychiatrist. The average length of stay in the program was 41 days. The average Neuropsychiatric Inventory score improved from 31 to 9.4 at discharge, and the number of medications decreased from 16 to 9. Among the 102 patients, 48 (47%) improved with VBM alone, avoiding hospital admission. In a survey of 60 LTC teams, 49 (81%) responded. Over 90% noted improvement in neuropsychiatric symptoms and expressed satisfaction with the quality of care and support provided by the VBM team. All respondents felt heard and more equipped to care for residents in-house, though some reported challenges with the virtual technology. These first-year insights into operations will inform continuous quality improvement. Further evaluation of caregiver satisfaction and the effectiveness of the NP-led model is warranted.

